# Cost-effectiveness of a non-pharmacological treatment vs. “care as usual” in day care centers for community-dwelling older people with cognitive impairment: results from the German randomized controlled DeTaMAKS-trial

**DOI:** 10.1007/s10198-020-01175-y

**Published:** 2020-03-26

**Authors:** Kathrin Steinbeisser, Larissa Schwarzkopf, Elmar Graessel, Hildegard Seidl

**Affiliations:** 1 Institute of Health Economics and Health Care Management, Helmholtz Zentrum München, German Research Center for Environmental Health, Ingolstädter Landstr. 1, 85764 Neuherberg, Germany; 2 IFT Institut für Therapieforschung, Leopoldstr. 175, 80804 Munich, Germany; 3 Department of Psychiatry and Psychotherapy, Center for Health Service Research in Medicine, University Hospital Erlangen, Friedrich-Alexander-University Erlangen-Nuremberg, Schwabachanlage 6, 91054 Erlangen, Germany; 4 Quality Management and Gender Medicine, München Klinik gGmbH, München Klinik Schwabing, Kölner Platz 1, 80804 Munich, Germany

**Keywords:** Dementia, MCI, Cost-effectiveness analysis, MMSE, ETAM, Non-pharmacological treatment, I12 Health Behavior

## Abstract

**Background:**

Cognitive impairment in older adults causes a high economic and societal burden. This study assesses the cost-effectiveness of the multicomponent, non-pharmacological MAKS treatment vs. “care as usual” in German day care centers (DCCs) for community-dwelling people with mild cognitive impairment (MCI) or mild to moderate dementia over 6 months.

**Methods:**

The analysis was conducted from the societal perspective alongside the cluster-randomized controlled, multicenter, prospective DeTaMAKS-trial with waitlist group design. Outcomes were Mini-Mental Status Examination (MMSE) and Erlangen Test of Activities of Daily Living in Persons with Mild Dementia or Mild Cognitive Impairment (ETAM) of 433 individuals in 32 DCCs. Incremental differences in MMSE and ETAM were calculated via a Gaussian-distributed and incremental cost difference via a Gamma-distributed Generalized Linear Model. Cost-effectiveness was assessed via cost-effectiveness planes and cost-effectiveness acceptability curves (CEAC).

**Results:**

At 6 months, MMSE (adjusted mean difference = 0.92; 95% confidence interval (CI): 0.17 to 1.67; *p* = 0.02) and ETAM (adjusted mean difference = 1.00; CI: 0.14 to 1.85; *p* = 0.02) were significantly better in the intervention group. The adjusted cost difference was − €938.50 (CI: − 2733.65 to 763.13; *p* = 0.31). Given the CEAC, MAKS was cost-effective for 78.0% of MMSE and 77.4% for ETAM without a need for additional costs to payers.

**Conclusions:**

MAKS is a cost-effective treatment to stabilize the ability to perform activities of daily living and cognitive abilities of people with MCI or mild to moderate dementia in German DCCs. Thus, MAKS should be implemented in DCCs.

## Background

Demographic change leads to an aging population and is expected to increase the prevalence of disability and chronic conditions such as cognitive impairment [1]. Cognitive impairment in older people often begins with mild cognitive impairment (MCI), which can be a transition stage to dementia with a conversion rate of about 15% per year [2]. Over the last 10 years, the prevalence of MCI in Germany for people older than 65 years was 13.0 to 20.0% [3, 4]. In 2017, more than 1.7 million people older than 65 years in Germany suffered from dementia with an incidence of 300,000 cases per year [5]. Owing to rising life expectancy, the prevalence of dementia is estimated to increase to 3 million cases in Germany by 2050 [6]. Cognitive impairment causes high economic and societal burden due to the high costs of care, especially for institutionalization [7–10].

To prevent institutionalization and minimize costs resulting from deterioration of cognitive impairment, adequate treatments are necessary for community-dwelling people with cognitive impairment. Until recently, the literature has mainly focused on pharmacological treatments for effective management strategies for cognitive impairment (e.g., [11–13]). However, the literature states that non-pharmacological treatments are useful and potentially cost-effective approaches to improve and stabilize people’s cognitive and functional abilities [14–17]. To affect multiple domains, a combination of cognitive and physical interventions (multimodal approaches) within non-pharmacological treatments is recommended [15, 18].

In Germany, different services exist for community-dwelling people with cognitive impairment. One service is the adult day care center (DCC), which is a regular service in many industrialized countries [19]. DCCs support the social, health, and daily living needs of people in need of care (including people with cognitive impairment) in a group setting during daytime hours and thus minimize informal caregivers’ burden of care during the day. DCCs are facilities located in or close to a community where older adults live. They enable community-dwelling older adults or people with disabilities or chronic diseases to remain living at home through providing a supportive environment regarding social needs and activities of daily living (ADLs), such as eating or going to the toilet. Furthermore, people with cognitive or physical health needs receive support through different health and occupational programs (e.g., promotion of physical activity through balloon-games). “Care as usual” in German DCCs is normally considered as assistance with daily activities like eating or going to the toilet, managing medication, and the offer of different types of occupational programs, such as playing board games. The scope of assistance is individual to every DCC. Especially the offer of occupational programs can be different regarding the scope and types of activities provided in the DCCs [20, 21]. Support is provided by formal caregivers, such as skilled nurses and occupational therapists [20, 22, 23]. Germany’s statutory nursing care insurance covers costs of day care including transportation for statutory-insured adults with a level of care (since 2017: "care grades"). Only costs for food and specific investments are not covered. The amount of financial support depends on the individual’s level of care; one is the level for the lowest level of assistance needed, while  three is the level for the highest assistance needed [22]. People applying for a level of care are evaluated for the amount of assistance they need by the statutory Health Insurance Medical Service. The prerequisites for receiving day care depend on the individual’s need and the availability of a caregiver during day [22, 23]. Independent from financing day care, similar models as above described “care as usual” day care exist in other industrialized countries [19, 24, 25].

According to previous research [19, 26, 27], DCCs show a positive effect on the well-being of older adults who visit DCCs regularly. To date, mainly clinical effectiveness of non-pharmacological treatments for community-dwelling people with cognitive impairments and their caregivers was assessed (e.g., [14, 16, 19, 25, 27, 28]). However, literature states that cost-effectiveness analyses focusing on evidence-based, structured, non-pharmacological treatments in the setting DCC for community-dwelling people with cognitive impairments continue to be limited [16, 27, 29–31]. Researchers suggest that future trials should systematically include cost-related measures [14, 27, 29]. Furthermore, Nagy et al. recommend that economic evaluations should include analyses of cognitive, as well as functional, parameters of people with cognitive impairment [13].

The objective of this study is to assess the cost-effectiveness of a multicomponent, non-pharmacological treatment vs. “care as usual” in DCCs for community-dwelling people with cognitive impairment from the societal perspective.

## Methods

### Study design

We conducted a cost-effectiveness analysis (CEA) alongside the cluster-randomized, controlled, multicenter, prospective DeTaMAKS-trial (German acronym for “*D*ementia in *D*ay care (German “*T*agespflege”) with *M*otor stimulation, *A*ctivities of daily living stimulation, *C*ognitive (German “*K*ognitiv”) stimulation, and *S*ocial functioning”). The treatment is called “MAKS”. The DeTaMAKS-trial had a waitlist control group design and was applied within 34 German DCCs between April 2014 and March 2017 [32].

Individuals in DCCs were included if they had MCI, mild or moderate dementia, and if informed consent was given. Individuals who were blind, deaf, without a caregiver, not able to communicate, or had suffered more than one stroke, severe depression, schizophrenia, an addictive disorder, had concrete plans for institutionalization, or were attending DCCs less than once a week were excluded [20]. All DCCs were randomized into two groups (intervention vs. “care as usual”). Further details on the recruitment strategy of DCCs and the eligibility criteria of DCCs and participants are described in detail elsewhere [28, 32]. All procedures were approved by the Friedrich Alexander University Erlangen-Nuremberg Ethics Committee. The trial’s registration number is ISRCTN16412551.

For the CEA, participants were assessed both at baseline (*t*_0_) and at 6-month follow-up (*t*_1_) of the intervention. Both the intervention group (IG) and the control group (CG) included only individuals who started the allocated treatment and did not die during the intervention phase (intention to treat (ITT)). A sensitivity analysis included all individuals in the IG and CG who completed the intervention as per protocol (complete cases).

### Intervention

The IG underwent the treatment “MAKS”, whereas the CG continued with “care as usual”. MAKS is a non-pharmacological, multicomponent, group-based treatment developed for patients in DCCs. The treatment’s aim is to improve or at least stabilize the ability to perform ADLs and cognitive abilities of people with MCI or mild to moderate dementia in German DCCs. MAKS combines four components (social warm-up session (S) (sensori)motor activation (M), cognitive stimulation (K), activation of ADLs (A)). Oswald et al. [33, 34], Olazarán et al. [14] and Özbe et al. [15] found multicomponent-interventions to be more effective than single-component interventions and that they generate broader positive outcomes. Thus, MAKS includes more than one component. According to the German “S3-Leitlinie Demenzen” [18] and the British “NICE-SCIE Guideline Dementia” [31], activities to stimulate cognition (K), improve or stabilize ADLs (A) and physical activity (M) are effective strategies to minimize risk factors for dementia in patients with MCI or to delay the disease’s progress in patients with mild to moderate dementia. Furthermore, the “social warm-up session” (S) was added to MAKS, because of former research stating social participation to minimize the risk of dementia [35–37]. The importance of social interactions to minimize the risk of dementia was pointed out by the systematic review of Kuiper et al. [38]. Additionally, NICE-SCIE recommends that e.g., “people with mild-to-moderate dementia of all types should be given the opportunity to participate in a structured group cognitive stimulation” [31].

The four components of MAKS are always applied in the same order, thus forming an intervention unit that lasts approximately 2 h per day. The daily intervention begins with a social warm-up session, such as a discussion about various topics or a group meditation. After that, a sensorimotor activation session follows, which addresses gross and fine motor skills, sensory perception, and balance. The cognitive stimulation session consists of game-based exercises, such as knowledge quizzes and memory games. The last session addresses the activation of ADLs through social tasks (e.g., baking, doing handicrafts). Social interaction is important in all sessions (e.g., completion of tasks together) [28, 32]. Further details of MAKS can be found elsewhere [32, 39].

MAKS’ clinical effectiveness was proven in the described randomized, controlled DeTaMAKS-trial [28]. The trial’s aim was to evaluate MAKS’ effect on cognitive abilities and capabilities to perform ADLs in people with MCI or dementia in German DCCs.

“Care as usual” within the DeTaMAKS-trial was defined as above described “care as usual” in German DCCs.

### Costs

The economic evaluation was performed from the societal perspective. All costs were calculated for the year 2014/2015 and reported in Euros.

Service utilization was assessed at *t*_0_ and *t*_1_ via proxy interviews with the participants’ informal caregivers. The assessment was based on a modified version of the validated FIMA questionnaire [40]. The reference period for *t*_0_ covered the 3-month period before *t*_0_. The reference period for *t*_1_ was the 6-month intervention period.

Costs for informal and formal care, as well as for therapeutic services, were calculated by applying the German unit costs of Bock et al. [41] and using several updated sources for 2014/2015 (e.g., [42–45]). Costs for informal care were calculated according to the opportunity cost approach [46]. All caregivers were asked about their amount of informal care time and whether they reduced their work to undertake caregiving. If so, work productivity loss was calculated by average wage rates per hour. Additional hours were calculated by average rates for leisure time per hour [41]. Further details on unit costs and their data sources can be found in Table 1.

**Table 1:** Cost categories of service utilization and unit costs in € for 2014/2015

Cost category	Unit	Unit costs in €	Source
**Costs of service utilization**			
**Formal care**			
Home nursing service	h	42.00	[41], updated
Paid service for household support	h	21.00	[41], updated
Service for supervision at home	h	31.44	[41], updated
Short-term care	day	55.35	[45]
Meal delivery	day	1.00	[43]
**Informal care**			
Care during leisure time	h	22.32	[41], updated
Work productivity loss due to caregiving	h	31.50	[41], updated
**Services provided for informal caregivers**			
Training in nursing skills	day	90.00	[47]
Consultation	h	40.00	[47]
Patient group supervision	Contact	25.00	[47, 48]
Self-help group sessions including patient supervision	Contact	14.33	[49, 50], average of salary and rental costs
**Therapeutic services**			
Physical therapy	Contact	17.45	[41], updated
Occupational therapy	Contact	39.34	[41], updated
Medical pedicure	Contact	29.75	[41], updated
** Intervention costs**			
MAKS training session	h	29.90	Wage/hour by University Hospital Erlangen
MAKS refresher course	h	29.90	Wage/hour by University Hospital Erlangen
Phone-based support	h	29.90	Wage/hour by University Hospital Erlangen
Travel costs of MAKS trainer	km	0.20	[51]
Hotel costs of MAKS trainer	Overnight stay	70.00	Average price of overnight stays at hotel [52]
Manual	Book	48.80	Retail price

*MAKS* non-pharmacological treatment (Motor stimulation, Activities of daily living stimulation, Cognitive stimulation, and Social functioning)

### Intervention costs

Intervention costs consisted of personnel costs for the MAKS trainer for providing onsite training and phone-based support for questions regarding the implementation of MAKS. Additionally, the trainer’s hotel and travel costs to the onsite sessions were considered. Furthermore, material costs for the manual provided to the DCCs were accounted for (see Table 1).

### Effects

The effect of MAKS on cognitive abilities was operationalized by the Mini-Mental Status Examination (MMSE) [53]. The effect on capabilities to perform ADLs was operationalized by the Erlangen Test of Activities of Daily Living in Persons with Mild Dementia and Mild Cognitive Impairment (ETAM) [54, 55]. MMSE and ETAM were both assessed at *t*_0_ and *t*_1_. Both tests have a range from 0 to 30 points with higher values indicating better performance.

### Statistical analysis

The economic evaluation included a CEA with MMSE and ETAM as the intervention’s effects. Both MMSE and ETAM were conducted on an ITT basis. All analyses were performed at an alpha-level of 0.05. To examine differences between IG and CG at *t*_0_, subject characteristics were compared using Pearson’s Chi square tests for independence for categorical variables and Mann–Whitney *U* tests for continuous variables.

To calculate the incremental difference of MMSE and ETAM between the IG and CG at *t*_1_, we used Gaussian-distributed Generalized Linear Models. For this analysis, we controlled for age, gender, MMSE, and ETAM at *t*_0_.

Costs were calculated by multiplying the reported utilization figures by their respective unit costs. Here, single missing items were assumed to be true zeros. For therapeutic services not being assessed at *t*_0_, multiple imputation was performed within the ITT population. Total costs were derived by summing up the costs of each cost domain. To estimate the incremental cost difference, we used a Gamma-distributed Generalized Linear Model to consider the right-skewed nature of cost data [56]. We assigned a small value of €10.00 for individuals without costs (IG: *n* = 2 at *t*_0_) to avoid them being excluded from the analyses. Cost differences adjusted for age, gender, and costs at *t*_0_ were estimated based on recycled predictions with group assignment (IG vs. CG) as the coefficient of interest. Recycled predictions create an identical covariate structure for both the IG and the CG. First, costs are predicted under the assumption that all individuals are cases, i.e. all individuals are in the IG. Subsequently, costs are predicted under the assumption that all individuals are controls, i.e. all individuals are in the CG, and predict costs. Calculating the difference in the mean predictions for all individuals between these two scenarios then results in an estimate of the adjusted marginal difference in costs between IG and CG [57]. For the adjusted cost difference, a 95% confidence interval (CI) was estimated from 1000 bootstrap replications using the percentile method. Similar to the previous analysis of MAKS’ clinical effectiveness [28], costs and effects were calculated on an individual-, rather than cluster-based structure to allow comparability.

For ETAM and MMSE, we analyzed incremental cost-effectiveness ratios (ICERs) when applicable (not negative) [58]. Simultaneous bootstrapping (*n* = 1000) of incremental cost and incremental effect estimates addressed estimation uncertainty. Those replications were plotted on the cost-effectiveness plane (CE plane). Furthermore, we calculated cost-effectiveness acceptability curves (CEAC) based on the resulting bootstrap distribution. Those CEACs indicate the likelihood that the intervention is cost-effective for a given value of willingness to pay.

**Fig. 1:**
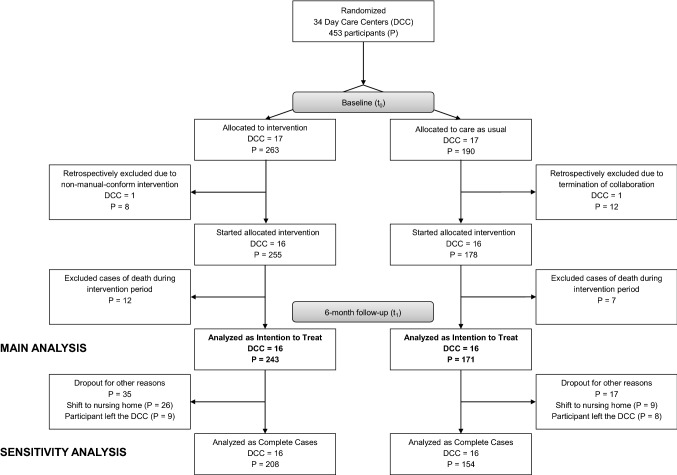
Flow diagram of the DeTaMAKS-trial’s study sample. *DCC* day care center, *P* participant

Missing values were assumed to be missing at random, which means that observed variables before dropout can be used to predict the missing value. It is supposed that there is no pattern of missingness and bias results to be small [59]. Missing values were imputed for those study participants with dropout reasons other than death (see Fig. 1). ETAM and MMSE were imputed using an expectation maximization algorithm. This method uses the variables that show the greatest correlation with the missing variable [28].

### Sensitivity analyses

Finally, we performed three sensitivity analyses (SA).

For SA_1_, we repeated all analyses within complete cases.

For SA_2_, intervention costs were calculated within the ITT population by applying a real-world situation for all costs of MAKS’ implementation.

As different approaches for costs for informal care exist, we also calculated costs for informal care according to the often-used proxy good method in the ITT population as SA_3_ [60, 61]. For this approach, we used the minimum gross wage including incidental wage costs for skilled nurses. For 2015, this value was €12.03 [62, 63].

All analyses were performed with SAS (Version 9.4, SAS Institute Inc., Cary, NC, USA).

## Results

### Study sample

Figure 1 presents the flow chart of the study sample. At *t*_0_, 34 DCCs were randomized into two groups. Two out of the 34 recruited DCCs were excluded for analysis (DCC_1_: terminated collaboration treatment, DCC_2_: treatment was not performed according to the instruction manual). Thus, the final study sample resulted in the remaining 32 DCCs with a total of 433 individuals (IG: *n* = 255, CG: *n* = 178). Owing to death between *t*_0_ and *t*_1_, 19 individuals had to be excluded for the CEA based on ITT. Thus, the CEA included 243 individuals in the IG and 171 in the CG.

The 19 dropouts were significantly older than individuals who remained in the ITT analysis (86.3 versus 81.4 years). All other values of dropouts were similar to those in the ITT analysis.

**Fig. 2:**
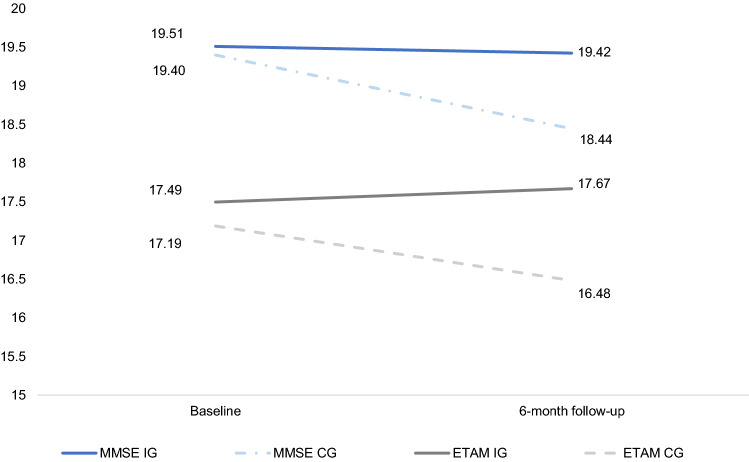
Changes in MMSE and ETAM between *t*_0_ and *t*_1_. *IG* intervention group, *CG* control group, *MMSE* Mini-Mental Status Examination, *ETAM* Erlangen Test of Activities of Daily Living in Persons with Mild Dementia or Mild Cognitive Impairment

The study sample’s baseline characteristics are shown in Table 2. Mean age was 81.4 years. Of 414 individuals, 259 (62.6%) were women (see Fig. 2). Randomization produced relatively well-balanced samples (see Table 2).

**Table 2:** Baseline characteristics of individuals stratified by group (*n* = 414)

		*N*	Total (*n* = 414)	Intervention group (58.7%) (*n* = 243)	Control group (41.3%) (*n* = 171)	*p* value
**Dementia patients**						
Age in years	Total	414	81.4 (7.7)	81.7 (7.9)	81.0 (7.4)	0.26^a^
Sex	Female	414	259 (62.6%)	152 (62.6%)	107 (62.6%)	1.00^b^
Education	Low (≤ 9 years)	413	317 (76.8%)	185 (76.5%)	132 (77.2%)	0.63^a^
	Middle (10–11 years)		51 (12.3%)	28 (11.6%)	23 (13.5%)	
	High (≥ 12 years)		45 (10.9%)	29 (12.0%)	16 (9.4%)	
Marital status	Married	414	169 (40.8%)	99 (40.7%)	70 (40.9%)	0.96^b^
	Widowed		221 (53.4%)	129 (53.1%)	92 (53.8%)	
	Divorced		12 (2.9%)	7 (2.9%)	5 (2.9%)	
	Single		12 (2.9%)	8 (3.3%)	4 (2.3%)	
Cognitive impairment (MMSE)	Total	414	19.5 (4.7)	19.5 (4.7)	19.4 (4.8)	0.68^a^
	24–30 (MCI)		89 (21.4%)	53 (21.8%)	36 (21.1%)	0.83^b^
	18–23 (mild dementia)		170 (41.1%)	102 (42.0%)	68 (39.8%)	
	10–17 (moderate dementia)		155 (37.4%)	88 (36.2%)	67 (39.2%)	
Activities of daily living (ETAM)	Total	414	17.4 (7.2)	17.5 (6.9)	17.2 (7.4)	0.71^a^
Care level	None	414	20 (4.8%)	8 (3.3%)	12 (7.0%)	0.27^b^
	Limited abilities in ADLs		46 (11.1%)	28 (11.5%)	18 (10.5%)	
	1 (low)		218 (52.7%)	136 (56.0%)	82 (48.0%)	
	2 (middle)		126 (30.4%)	69 (28.4%)	57 (33.3%)	
	3 (high)		4 (1.0%)	2 (0.8%)	2 (1.2%)	
Antidementia drugs	Total		122 (2.5%)	72 (29.8%)	50 (29.2%)	0.91^a^
Social behavior (NOSGER)	Total	414	15.6 (4.4)	15.5 (4.3)	15.7 (4.5)	0.48^a^
Neuropsychiatric symptoms (NPI-Q)	Total	412	5.4 (2.7)	5.3 (2.7)	5.4 (2.8)	0.83^a^
**Caregivers**						
Age in years	Total	414	59.6 (11.6)	59.5 (11.7)	59.7 (11.4)	0.76^a^
Sex	Female	414	303 (73.2%)	174 (71.6%)	129 (75.4%)	0.39^b^
Education	Low	414	166 (40.1%)	96 (39.5%)	70 (40.9%)	0.36^a^
	Middle		149 (36.0%)	83 (34.2%)	66 (38.6%)	
	High		99 (23.9%)	64 (26.3%)	35 (20.5)	
Employment status	Employed	414	226 (54.6%)	133 (54.7%)	93 (54.4%)	0.94^b^
Marital status	Married/long-term partnership	414	326 (78.4%)	187 (77.0%)	139 (81.3%)	**0.04**^b^
	Widowed		15 (3.6%)	12 (4.9%)	3 (1.8%)	
	Divorced		38 (9.2%)	18 (7.4%)	20 (11.7%)	
	Single		35 (8.5%)	26 (10.7%)	9 (5.3%)	
Relationship to person cared for	Spouse	414	112 (27.1%)	63 (25.9%)	49 (28.7%)	0.54^b^
	Daughter/son (in law)		277 (67.0%)	163 (67.1%)	114 (66.7%)	
	Other		25 (6.0%)	17 (7.0%)	8 (4.7%)	
Caregiver burden (BSFC-s)		414	12.7 (8.1)	12.2 (8.2)	13.4 (7.8)	0.08^a^
**Care status**						
Main caregiver	Yes	414	365 (88.2%)	210 (86.4%)	155 (90.6%)	0.19^b^
Main caregiver = only informal caregiver	Yes	414	186 (44.9%)	110 (45.3%)	76 (44.4%)	0.64^b^
Living together in same home	Yes	414	253 (61.1%)	139 (57.2%)	114 (66.7%)	**0.05**^b^
Duration of informal care in months	Total	413	59.8 (51.0)	58.7 (48.3)	61.2 (54.6)	0.79^a^
No. of visits/week to DCC within first month	Total	414	2.27 (1.3)	2.29 (1.3)	2.25 (1.2)	1.00^a^
Informal care time in hours per day	Total	414	3.2 (2.0)	3.1 (2.0)	3.3 (2.1)	0.40^a^

*MMSE* Mini-Mental Status Examination, *MCI* mild cognitive impairment, *ETAM* Erlangen Test of Activities of Daily Living in Persons with Mild Dementia or Mild Cognitive Impairment, *ADLs* activities of daily living, *NOSGER* Nurses’ Observation Scale for Geriatric Patients, social behavior subscale, *NPI-Q* Neuropsychiatric Inventory Questionnaire (number of symptoms), *BSFC-s* Burden Scale for Family Caregivers, short version, *DCC* day care center

Bold numbers: significant at *p* ≤ 0.05

Data presented as *n* (%)/mean (standard deviation) | any discrepancies in percentages due to rounding

^a^Based on Mann–Whitney *U* test, ^b^based on Pearson’s Chi square test

For SA_1_, we included 208 individuals in the IG and 154 individuals in the CG who completed the intervention. Similar to the ITT analysis, mean age was 81.3 years and 221 (61.0%) were women.

### Effects

Whereas unadjusted MMSE values at *t*_0_ were comparable between IG (19.51; SD = 0.30) and CG (19.40; SD = 0.36), they differed at *t*_1_: MMSE in the IG remained almost at the same level (19.42; SD = 0.37), MMSE in the CG declined (18.44; SD = 0.46). The adjusted difference was significant (adjusted mean difference = 0.92; CI: 0.17 to 1.67; *p* = 0.02).

Similar, ETAM at *t*_0_ started at a comparable level. The unadjusted value for the IG was 17.49 (SD = 0.44) and for the CG 17.19 (SD = 0.58). At *t*_1_, ETAM in the IG increased to 17.67 (SD = 0.44). In contrast, ETAM in the CG declined to 16.48 (SD = 0.63). The adjusted difference was significant (adjusted mean difference = 1.00; CI: 0.14 to 1.41; *p* = 0.02) (see Fig. 2).

### Service utilization and costs

Mean service utilization at *t*_0_ and *t*_1_ and mean costs per patient are presented in Table 3. At *t*_0_, individuals in the IG (€8551.57; SD = 5411.60) created similar unadjusted costs to those in the CG (€8089.63; SD = 4872.46). Costs for informal care were the largest contributor to costs of service utilization (84.9%).

**Table 3:** Mean service utilization in number of contacts and mean costs in € per individual for *t*_0_ and *t*_1_

Cost category	Unit	Intervention group (58.7%) (*n* = 243)				Control group (41.3%) (*n* = 171)			
		Mean service utilization (SD)		Mean costs (SD)		Mean service utilization (SD)		Mean costs (SD)	
		*t*_0_^a^	*t*_1_^b^	*t*_0_	*t*_1_	*t*_0_^a^	*t*_1_^b^	*t*_0_	*t*_1_
**Costs of service utilization**									
** Formal care**				**1131.91 (1466.13)**	**2513.83 (3008.99)**			**906.83 (1198.01)**	**2070.40 (2514.34)**
Home nursing service	h	17.44 (2.96)	36.92 (64.49)	680.09 (1179.26)	1598.71 (2615.99)	11.31 (20.04)	26.72 (50.50)	479.13 (845.30)	1138.17 (2089.16)
Paid service for household support	h	10.15 (29.13)	21.47 (59.09)	221.80 (637.88)	484.62 (1222.10)	10.53 (22.38)	17.87 (41.63)	219.79 (464.11)	385.81 (849.87)
Service for supervision at home	day	1.78 (6.03)	4.35 (15.11)	75.92 (271.96)	146.10 (469.37)	2.53 (8.96)	6.05 (17.71)	56.48 (192.29)	176.84 (460.17)
Short-term care	day	2.75 (7.84)	4.67 (11.20)	146.73 (435.15)	241.96 (542.48)	2.56 (6.13)	7.45 (15.70)	146.13 (343.81)	388.56 (848.52)
Meal delivery	day	7.28 (20.49)	9.79 (31.80)	7.37 (20.22)	11.71 (32.25)	6.09 (18.89)	12.68 (34.35)	5.31 (17.49)	11.75 (32.62)
** Informal care**				**6962.63 (4919.18)**	**13,895.35 (10,503.54)**			**7499.85 (4952.01)**	**16,200.71 (11,330.74)**
Care during leisure time	h	252.89 (179.15)	515.50 (439.87)	6187.17 (4113.42)	12,523.37 (9933.37)	264.29 (191.18)	554.23 (414.93)	6401.02 (3736.34)	13,974.66 (9244.08)
Work productivity loss due to caregiving	h	25.31 (69.51)	42.8 (122.86)	775.46 (2174.98)	1371.98 (3756.65)	33.62 (82.46)	67.74 (172.51)	1098.83 (2652.27)	2226.05 (5428.98)
**Services provided for informal caregivers**				**53.27 (143.01)**	**169.78 (430.78)**			**52.68 (157.10)**	**97.45 (237.06)**
Training in nursing skills	day	–	0.02 (0.14)	–	2.34 (12.24)	–	0.03 (0.18)	–	3.26 (15.73)
Consultation	Contact	0.37 (1.29)	0.84 (3.01)	32.07 (108.61)	83.10 (239.12)	0.34 (1.49)	0.58 (2.37)	27.16 (121.66)	50.65 (188.05)
Self-help group sessions incl. patient supervision	Contact	0.23 (1.35)	0.53 (3.09)	3.57 (20.28)	9.51 (43.62)	0.88 (3.93)	0.97 (4.91)	2.83 (9.43)	13.30 (52.81)
Patient group supervision	Contact	0.68 (2.52)	2.63 (12.75)	17.63 (62.94)	74.83 (315.02)	0.19 (0.64)	0.86 (3.75)	22.69 (100.73)	30.32 (123.65)
**Therapeutic services**					**243.77 (527.41)**				**188.92 (447.63)**
Physical therapy	Contact	–	7.13 (16.31)	–	132.81 (280.24)	–	6.30 (15.06)	–	113.78 (259.73)
Occupational therapy	Contact	–	2.42 (9.15)	–	110.68 (357.15)	–	1.64 (8.11)	–	74.24 (318.25)
Medical pedicure	Contact	–	0.00 (0.00)	–	0.00 (0.00)	–	0.04 (0.40)	–	0.90 (9.37)
**Intervention costs**		–			**15.34**	–	–	–	–
MAKS training session	h	–			3.94	–	–	–	–
MAKS refresher course	h	–			1.97	–	–	–	–
Phone-based support	h	–			1.97	–	–	–	–
Travel costs of MAKS trainer	km	–			3.13	–	–	–	–
Hotel costs of MAKS trainer	Overnight stay	–			1.15	–	–	–	–
Manual	Book	–			3.19	–	–	–	–
**Total costs**^c^				**8089.63 (4871.46)**	**16,359.44 (10,333.29)**			**8551.57 (5411.60)**	**18,526.82 (11,374.81)**

Data presented as mean (standard deviation), any discrepancies due to rounding

Single missing items in resource utilization of complete cases not imputed, single missing items in cost calculation for complete cases assumed to be true zeros; thus, slightly different results due to multiplication of unit costs with mean service utilization

Bold numbers indicates summed costs of each category *MAKS* non-pharmacological treatment (Motor stimulation, Activities of daily living stimulation, Cognitive stimulation, and Social functioning)

^a^Reference period: 3-month period before *t*_0_, ^b^reference period: 6-month intervention period, ^c^imputed values, summing of distinct cost categories yields slight deviation

At *t*_1_, adjusted total costs resulted in lower costs in the IG of − €938.50 (CI: − 2733.65 to 763.13; *p* = 0.31). Except for informal care, the IG incurred higher costs than the CG in all other categories. For informal care, we observed − €1159.63 (CI: − 3078.81 to 786.73; *p* = 0.25) lower costs in the IG. However, in none of the categories was the cost difference statistically significant. Detailed information about adjusted costs can be found in Table 4.

**Table 4:** Adjusted costs and cost differences in € for *t*_1_ per individual

	Intention to treat analysis			
	Intervention group [95% CI]	Control group [95% CI]	Cost difference [95% CI]	*p* value
**Total costs**	**17,169.52 [15,938,52; 18,472.36]**	**18,108.01 [16,731.65; 19,642.09]**	**− 938.50 [− 2733.65; 763.13]**	0.31
Formal care	2519.50 [2200.25; 2849.82]	2288.87 [1929.27; 2709.91]	230.63 [− 200.43; 654.13]	0.28
Informal care	14,636.34 [13,299.19; 16,229.85]	15,795.86 [14,441.91; 17,327.65]	− 1159.63 [− 3078.81; 786.73]	0.25
Services provided for informal caregiver	167.96 [115.44; 240.66]	114.65 [76.22; 181.20]	53.30 [− 2.69; 115.49]	0.06
Therapeutic services	239.59 [117.37; 308.27]	164.95 [111.80; 222.95]	74.63 [− 10.25; 156.16]	0.07

	Complete case analysis (sensitivity analysis 1)			
	Intervention group [95% CI]	Control group [95% CI]	Cost difference [95% CI]	*p* value
**Total costs**	**17,755.30 [16,362.74; 19,399.73]**	**18,247.59 [16,759.36; 19,272.96]**	**− 492.29 [− 3389.92; 2465.11]**	0.65
Formal care	2549.60 [2190.30; 2956.71]	2216.87 [1844.48; 2618.74]	332.73 [− 141.77; 789.61]	0.16
Informal care	15,145.71 [13,532.91; 16,830.79]	15,953.54 [14,360.91; 17,524.87]	− 807.28 [− 2880.75; 1408.10]	0.47
Services provided for informal caregiver	116.01 [113.37; 237.59]	115.44 [75.73; 167.81]	50.58 [− 12.49; 119.26]	0.12
Therapeutic services	258.13 [188.71; 336.05]	176.55 [121.32; 243.81]	81.58 [− 13.73; 174.60]	0.08

All cost estimates except for informal care based on two-part model

*95% CI* 95% confidence interval

### Intervention costs

Four MAKS training sessions of 8 h for a pool of four DCCs with three participating employees per DCC were proposed (total costs: €956.80). The MAKS refresher courses were planned for a pool of four DCCs with a total of four sessions per course (total costs: €478.40). For every DCC, one manual was considered in the intervention’s cost calculation (total costs: €774.40). A total of 3800 km (total costs: €760.00) and four hotel overnight stays (total costs: €280.00) were planned for the MAKS trainer. The ITT analysis resulted in total mean intervention costs of €15.34 per patient or €233.00 per DCC.

### Cost-effectiveness

**Fig. 3:**
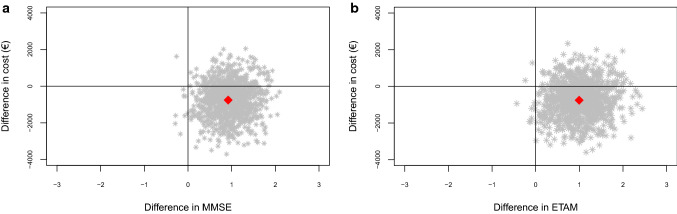
**a** Intention to treat analysis: cost-effectiveness plane for the difference in MMSE at *t*_1_. **b** Intention to treat analysis: cost-effectiveness plane for the difference in ETAM at *t*_1_. *MMSE* Mini-Mental Status Examination, *ETAM* Erlangen Test of Activities of Daily Living in Persons with Mild Dementia or Mild Cognitive Impairment

Figure 3a shows the CE plane of MMSE, Fig. 3b of ETAM. For both MMSE (76.7%) and ETAM (77.1%), most of the cost-effect pairs were located in the south-east quadrant of the CE plane. This quadrant suggests better effects and fewer costs. Although the intervention costs have been included, overall costs were lower in the IG (Table 4). In the north-east quadrant, 22.4% of MMSE and 21.8% of ETAM replications were located. This quadrant suggests better effects but higher costs.

**Fig. 4:**
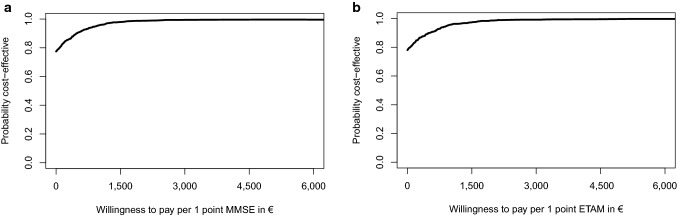
**a** Intention to treat analysis: cost-effectiveness acceptability curve for the difference in MMSE at *t*_1_.** b** Intention to treat analysis: cost-effectiveness acceptability curve for the difference in ETAM at *t*_1_. *MMSE* Mini-Mental Status Examination, *ETAM* Erlangen Test of Activities of Daily Living in Persons with Mild Dementia or Mild Cognitive Impairment

Given the CEAC (Fig. 4a, b), MAKS was cost-effective for 78.0% of MMSE and 77.4% for ETAM replications in comparison with “care as usual” without a need for additional costs to payers (willingness to pay of €0.00). Probability of 95.0% of acceptable cost-effectiveness was reached for a maximum willingness to pay of €939.66 for MMSE and €937.73 for ETAM. All ICERs resulted in negative values and thus were not reported.

### Sensitivity analyses

#### SA_1_: complete case analysis

Similar to the ITT analysis, MMSE (adjusted mean difference = 1.08; CI: 0.25 to 1.91; *p* = 0.01) and ETAM (adjusted mean difference = 1.14; CI: 0.19 to 2.10; *p* = 0.02) in SA_1_ showed significantly better results in the IG than in the CG. Owing to less intervention utilization, the SA_1_ analysis resulted in slightly fewer total mean intervention costs than the ITT analysis (€14.63/patient, €190.13/DCC). Only two DCCs took advantage of the MAKS refresher course. Thus, only two instead of four sessions took place, and the costs for travelling and overnight stays, as well as for trainer wages, were lower. Furthermore, the phone-based support could be managed within approximately 0.5 h instead of the initially assumed 1 h per DCC.

Similar to the ITT analysis, adjusted total costs at *t*_1_ resulted in lower costs in the IG of − €492.29 (CI: − 3389.92 to 2465.11; *p* = 0.65). Equally, only informal care resulted in lower costs in the IG. None of the cost differences was statistically significant (see Table 4).

**Fig. 5:**
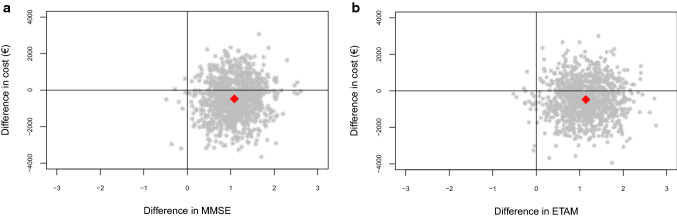
**a** Sensitivity analysis 1: cost-effectiveness plane for the difference in MMSE at *t*_1_. **b** Sensitivity analysis 1: cost-effectiveness plane for the difference in ETAM at *t*_1_

Within SA_1_, 67.5% of MMSE and 65.1% of ETAM were located in the south-east quadrant of the CE plane (Fig. 5a, b). In the north-east quadrant, 31.7% of MMSE and 33.0% of ETAM replications were located in the north-east quadrant.

**Fig. 6:**
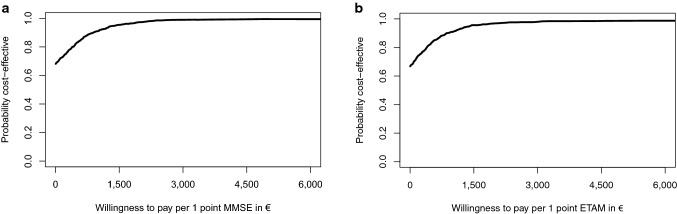
**a** Sensitivity analysis 1: cost-effectiveness acceptability curve for the difference in MMSE at *t*_1_. **b** Sensitivity analysis 1: cost-effectiveness acceptability curve for the difference in ETAM at *t*_1_

Given the CEAC (Fig. 6a, b), MAKS was cost-effective for 68.5% of MMSE and 66.8% for ETAM replications in comparison with “care as usual” without a need for additional costs to payers.

#### SA_2_: real-world situation

SA_2_ based on the ITT population. Therefore, effects were expected to be similar to the ITT analysis. For SA_2_, the planned total mean intervention costs (€960.00/DCC) will be higher than in the ITT analysis. The higher costs will be caused by the extension of MAKS sessions from 8 h up to 16 h. Furthermore, the MAKS refresher course will be mandatory for every DCC (ITT and SA_1_: voluntary) with a course fee of €290.00 and three required participants per DCC. Additionally, the printed manual will be converted into an online tool and has to be purchased for €90.00.

**Fig. 7:**
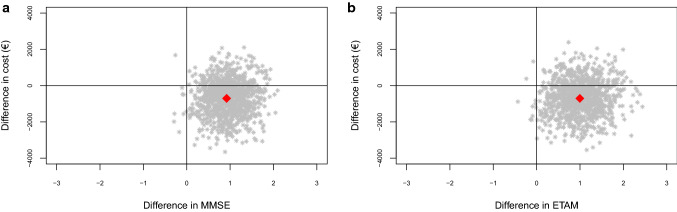
**a** Sensitivity analysis 2: cost-effectiveness plane for the difference in MMSE at *t*_1_. **b** Sensitivity analysis 2: cost-effectiveness plane for the difference in ETAM at *t*_1_

Similar to the ITT analysis, in SA_2_ 74.7% of MMSE and 75.6% of ETAM of the cost-effect pairs were in the south-east quadrant of the CE plane (Fig. 7a, b). For MMSE, 24.4% of the replications were in the north-east quadrant, and 23.4% for ETAM.

**Fig. 8:**
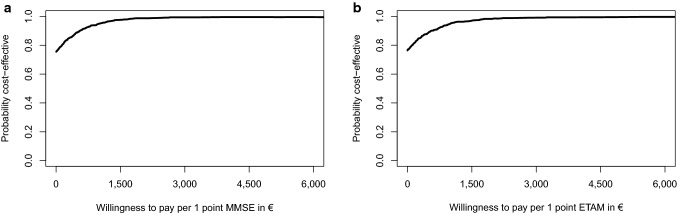
**a** Sensitivity analysis 2: cost-effectiveness acceptability curve for the difference in MMSE at *t*_1_. **b** Sensitivity analysis 2: cost-effectiveness acceptability curve for the difference in ETAM at *t*_1_

Given the CEAC (Fig. 8a, b), MAKS was cost-effective for 75.5% of MMSE and 76.4% for ETAM replications in comparison with “care as usual” without a need for additional costs to payers.

#### SA_3_: proxy good approach for costs of informal care

Table 5 shows the adjusted costs and cost differences in € for *t*_1_ per individual according to the proxy good approach. Similar to the opportunity cost approach, adjusted total costs in SA_3_ resulted in lower costs in the IG. For informal care, we observed − €661.21 (CI: − 1399.33 to 251.33; *p* = 0.2) lower costs in the IG than in the CG. However, cost difference was not statistically significant.

**Table 5:** Sensitivity analysis 3: adjusted costs and cost differences in € for *t*_1_ per individual in the intention to treat population according to proxy good approach

	Intervention group [95% CI]	Control group [95% CI]	Cost difference [95% CI]	*p* value
**Total costs**	**10,359.67 [9843.59; 10,730.98]**	**10,902.48 [9980.98; 11,787.83]**	**− 542.82 [− 1612.05; 585.14]**	0.2
Informal care	7678.79 [7142.19; 8021.48]	8340.00 [7508.83; 8995.08]	− 661.21 [− 1399.33; 251.33]	0.2

*95% CI* 95% confidence interval. Costs for informal care were calculated with €12.03. Other cost domains equal to Table 4

**Fig. 9:**
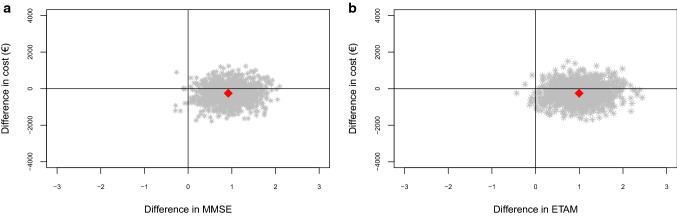
**a** Sensitivity analysis 3: cost-effectiveness plane for the difference in MMSE at *t*_1_. **b** Sensitivity analysis 3: cost-effectiveness plane for the difference in ETAM at *t*_1_

Similar to the ITT analysis, in SA_3_ 67.3% of MMSE and 66.3% of ETAM of the cost-effect pairs were in the south-east quadrant of the CE plane (Fig. 9a, b). For MMSE, 31.8% of the replications were in the north-east quadrant, and 32.7% for ETAM.

**Fig. 10:**
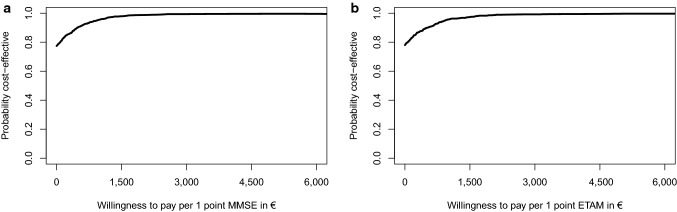
**a** Sensitivity analysis 3: cost-effectiveness acceptability curve for the difference in MMSE at *t*_1_. **b** Sensitivity analysis 3: cost-effectiveness acceptability curve for the difference in ETAM at *t*_1_

Given the CEAC (Fig. 10a, b), MAKS was cost-effective for 77.4% of MMSE and 78.0% for ETAM replications in comparison with “care as usual” without a need for additional costs to payers.

## Discussion

### Main findings and interpretation

This study investigated the cost-effectiveness of a non-pharmacological treatment in DCCs over a 6-month intervention period. To the knowledge of the authors, this is the first study to examine whether a structured non-pharmacological treatment in DCCs is cost-effective in comparison with “care as usual” in DCCs to improve or at least stabilize the ability to perform ADLs and the cognitive abilities of people with MCI or mild to moderate dementia. Adjusted costs at *t*_1_ in the IG were estimated at €17,169.52 (CI: 15,938.52 to 18,472.36), and in the CG at €18,108.01 (CI: 16,731.65 to 19,642.09) per individual. CEACs show that the intervention was cost-effective for 78.0% of bootstrapped MMSE and for 77.4% of bootstrapped ETAM replications in comparison with “care as usual” without a need for additional costs to payers. Sensitivity analyses supported our findings.

MMSE and ETAM both remained stable between *t*_0_ and *t*_1_ in the IG, whereas the values in the CG declined. Similar to other non-pharmacological treatments for older individuals with MCI or dementia, the slowing of decline in cognitive and physical functioning can be seen as effective [14, 64, 65]. This is also relevant in terms of clinical relevance. Without any intervention, a median decline of − 2.8 MMSE points per year, thus − 1.4 points in 6 months, in patients with dementia was observed in relevant studies and can be seen as a clinically meaningful decline [66, 67]. This is also confirmed by Howard et al. [68]. Andrews et al. [69] analyzed the “clinical meaningful decline” in people with dementia to lie between − 1 to − 3 MMSE-points. They additionally identified scores for “no meaningful decline” for different stages of disease severity. The researchers concluded that for people with mild cognitive impairment, “no meaningful decline” is considered as a decline less than or equal to − 0.19, for mild dementia − 0.40, and for moderate to severe dementia − 0.47. For DeTaMAKS we analyzed a pooled sample consisting of several stages of severity. Thus, we considered the lowest threshold reported by Andrews et al. (i.e. − 0.19) as the threshold for stable cognitive abilities [69]. The CG declined by − 0.96 MMSE-points between *t*_0_ and *t*_1_. Considering that individuals in the CG received some interventions and thus were more active than community-dwelling people without day care, this decline can be seen as a clinically meaningful decline. In contrast, the difference between *t*_0_ and *t*_1_ in the IG was only − 0.09 MMSE-points. Thus, no clinically meaningful decline could be detected, which underlines the clinical effectiveness of MAKS.

Internationally accepted thresholds for ETAM-decline are still lacking. Since we observed an increase of 0.18 ETAM-points in the IG, we concluded that capabilities to perform activities of daily living remained at least stable in the IG. This supports the thesis that MAKS is a clinically meaningful intervention. In contrast, the CG declined by − 0.71 ETAM-points. This suggests a—potentially clinically meaningful—decline.

The most important cost driver in the DeTaMAKS-trial was informal care. It has to be stated that inconsistency exists about the assessment of informal care costs. It is difficult to measure the exact time caregivers spend on supporting those in need of care. Furthermore, various methods exist to calculate costs. Whereas D’Amico et al. [70] calculated costs using minimum wages per hour, we calculated costs using average rates per hour in our main analysis. This approach is a common one in Germany and is based on current evidence [41]. We also confirmed our results through SA_3_, based on the proxy good method through using the minimum gross wage for skilled nurses. The different cost approaches have to be considered within comparison of the literature. However, studies on non-pharmacological treatments conducted from a societal perspective confirm that the main cost driver in community-dwelling people with cognitive impairment is informal care [8, 70, 71]. This is also in line with the assessment of general costs in health care caused by individuals with dementia [72, 73]. Regarding demographic change, interventions such as MAKS to stabilize older individuals’ health and thus reduce the burden on informal caregivers are highly recommended.

MAKS’ intervention costs of €15.34 per participant for the 6-month intervention period were cheap. Other non-pharmacological treatments with objectives similar to MAKS (comparison of £ with € for unit cost years adapted in studies) have higher intervention costs for the mentioned intervention periods [70, 74]. D’Amico et al. calculated £623.00 per participant for a 6-month intervention period, Knapp et al. [74] £220.50 per participant for a 7-week intervention period. Both the interventions of D’Amico et al. [70] (approximately £32.00/session per individual in community, 5 participants/session, costs for 2011) and Knapp et al. [74] (approximately £15.75/session per participant in care home or community, 5 participants/session, costs for 2001) were held twice per week. As the average number of DCC visits per week within the DeTaMAKS-trial’s IG was 2.29 times, intervention participation of twice per week per individual with an average of seven study participants per session was assumed. This was similar to the studies mentioned above. The low intervention costs resulted from its well-structured and sustainable approach. We trained skilled nursing staff to conduct the intervention within the DCCs. In contrast, the intervention sessions of Knapp et al. [74] and D’Amico et al. [70] were conducted by external researchers or facilitators with the assistance of skilled nurses at the community centers or care homes. This approach resulted in higher intervention costs due to higher personnel costs. Regarding the costs and sustainability of the intervention, this is a disadvantage in comparison to MAKS due to higher costs and the difficulty of continuing the intervention after finishing the study. In contrast, MAKS could be conducted exclusively by skilled nurses after intense training. Skilled nurses are highly qualified professionals who have the knowledge and experience of how to treat people with cognitive impairment, how to conduct non-pharmacological treatments, and also how to consider the patients’ current health status. Furthermore, they are familiar with the day-to-day structure in the DCCs they work in and are able to integrate MAKS’ activities appropriately. Instead of conducting “care as usual”, trained nurses working in DCCs can conduct the cost-effective intervention MAKS. Therefore, MAKS’ intervention costs do not cause additional personnel costs in comparison to “care as usual” (sunk costs) [75]. To guarantee the sustainability of an intervention, it is of great importance that it can be easily implemented into normal day-to-day structures. Further explanations for the lower costs of MAKS are the setting “DCC” and the low material costs. Whereas D’Amico et al. [70] had to plan costs for participants’ transport to a community center for the community-dwelling individuals, participants in the DeTaMAKS-trial caused no intervention-related travel costs. Additionally, DCCs normally have materials provided for activities (e.g., beads, balloons) within “care as usual”. Materials needed for MAKS are similar. Therefore, alongside the manual, no additional material costs for MAKS were assumed in comparison to “care as usual”.

Overall, findings on the cost-effectiveness of non-pharmacological interventional studies in older community-dwelling individuals with MCI or dementia are inconsistent and there is still a lack of evidence [30, 76]. Possible explanations for the inconsistencies can be the focus on different outcome parameters, sample sizes, or intervention periods. Additionally, many studies have adopted the narrower perspective of the health care and social system, instead of the comprehensive societal perspective [30]. Moreover, generalizability is restricted on account of different health care systems in other countries [30]. Furthermore, for previous studies about similar multicomponent, non-pharmacological treatments, no cost-effectiveness analyses are available [14, 77, 78]. For these reasons, comparability of our study with others is limited.

Our results showed that MAKS is cost-effective in stabilizing cognitive abilities and capabilities to perform ADLs. To assess cognitive abilities, tests such as “MMSE” or the “Alzheimer’s Disease Assessment Scale—Cognitive Subscale” (ADAS-Cog) are common methods. Whereas ADAS-Cog in its original version is used to assess cognitive function for patients with dementia only [79], MMSE is also used for patients with MCI [80]. However, comparable studies assessing the cost-effectiveness of non-pharmacological treatments addressed patients with dementia only. Therefore, it is likely that our results show slightly better cost-effectiveness because of the better health situations of individuals with MCI. The lack of studies examining the cost-effectiveness of non-pharmacological studies for individuals with MCI emphasizes the importance of our study.

Similar to our findings, D’Amico et al. [70] stated that cognitive stimulation therapy in comparison with “care as usual” assessed by MMSE was cost-effective at a low willingness to pay threshold. Similar to our study, the intervention period was 6 months. The main analysis was conducted from the health care and social perspective. However, a sensitivity analysis from a societal perspective could not confirm the results. It has to be noted that the study was conducted within nine care homes and nine community centers. The different settings cause different service utilization costs (e.g., no informal costs within care homes) than our study, which restricts comparisons to trends only. Knapp et al. [74] evaluated the cost-effectiveness of a cognitive stimulation therapy in 18 care homes and five DCCs. In line with our results, cost-effectiveness was shown for MMSE for a range of values of willingness to pay in a CEAC. However, detailed comparison is not possible because of a different perspective (health and personal social service), setting (majority: care homes), study participants (mild to moderate dementia only), and a shorter intervention period (7 weeks). As costs for individuals in community settings differ from those in care homes [8], we suggest conducting larger cost-effectiveness studies for each setting specifically. This would allow the detection of specific cost drivers and comparability with future cost-effectiveness studies.

To assess capabilities to perform ADLs, a variety of assessment tools exists. For example, the “Alzheimer’s Disease Cooperative Study—Activities of Daily Living Inventory” (ADCS-ADL) [81] was developed to assess abilities to perform ADLs in people with dementia. In D’Amico [70], ADCS-ADL was cost-effective from a health and social care, as well as from a societal perspective. Our study revealed similar results. However, ADCS-ADL and other tests assessing abilities to perform ADLs (e.g., Bristol Activities of Daily Living Scale, Bayer Activities of Daily Living Scale) are mainly observer rating scales and focus on assessing dementia. One of the main disadvantages of observer rating scales is rater bias, which can result in underestimating deficits in ADLs [82]. Therefore, we assessed our outcome through ETAM. ETAM is a brief, validated performance test to determine capabilities to perform ADLs in MCI or mild to moderate dementia. It is based on the International Classification of Functioning and Health and—in contrast to other tests—shows only moderate correlation coefficients with cognitive abilities [54, 55]. ETAM allowed us to correctly assess capabilities to perform ADLs in our study group via blind testers. Thus, comparability in future studies with similar designs will be facilitated.

We are not aware of current evidence on health care decision makers’ willingness to pay for non-pharmacological treatments such as MAKS. However, our results show that MAKS is cost-effective for a low willingness to pay. Still, further studies are needed to allow concrete comparability.

### Strengths and limitations

Major strengths of our study are the randomized design and the relatively large sample size in comparison to former studies with similar designs [30, 70, 74].

The detailed coverage of relevant costs allowed us to estimate MAKS’ impact from a societal perspective. This approach is recommended for cost-effectiveness analyses in dementia care by Wimo et al. [83] in order to include all relevant costs.

Unlike other cost-effectiveness studies, which mainly targeted individuals with dementia [30, 71, 74, 76, 84], we included individuals with MCI and dementia. MCI can often be a transition stage to dementia and should be targeted in more interventions in order to implement strategies to minimize the prevalence of dementia [2]. Furthermore, there is only sparse evidence about the cost-effectiveness of non-pharmacological treatments for individuals with MCI [30]. Therefore, our study contributed to an important topic.

Another strength of our study is the inclusion of three sensitivity analyses. The analyses support our findings and state that, even under different circumstances, MAKS is cost-effective for cognitive abilities and capabilities to perform ADLs.

According to the literature, external validity should be considered in interventional studies [85]. To address this issue, it is essential to mention that our study sample comprised 32 different DCCs all over Germany. Additionally, they were randomized into two groups. Therefore, MAKS is likely to be cost-effective in other German DCCs.

Some limitations of the present study have to be acknowledged. First, information on service utilization was based on self-reports. Therefore, it might be susceptible to recall bias. However, literature states that self-reports are a valid strategy to collect data on service utilization in the health care sector [86].

Another limitation of non-pharmacological studies is the restricted realization of blinding, which can lead to data collection bias. We could not blind therapists or participants as MAKS was a “visible treatment”. However, the evaluation of the outcomes was done by external testers blinded for intervention.

Internal validity might be affected by attrition through “shift to nursing home” (IG: *n* = 26, CG: *n* = 9). Our imputation approach included the observed variables before dropout that had a significant influence on costs. Thus, differences between IG and CG which already consisted at *t*_0_ were considered. If there was a decline in cognitive impairment caused by the intervention itself which would have led to “shift to nursing home”, imputation would not have prevented bias.

Finally, our study is limited to a 6-month intervention period to ensure attractiveness for study participation of DCCs for both the IG, as well as waitlist CG. Pre-study negotiations with DCCs found that a longer intervention period would have been unattractive for DCCs allocated to the waitlist CG. Owing to the waitlist control group design, no long-term effects could be analyzed. However, in comparison with other economic evaluations with similar study designs, the intervention period of 6 months can be seen as average. According to the systematic review by Nickel et al. [30], out of nine randomized controlled trials primarily focusing outcomes on individuals with MCI or dementia, five had a period for cost analysis of 6 or fewer months. To examine the long-term effects on service utilization and costs, future economic evaluations should include longer periods for cost analysis.

## Conclusions

In conclusion, our results emphasize that the non-pharmacological treatment MAKS is a cost-effective intervention to stabilize the ability to perform ADLs and the cognitive abilities of people with MCI or mild to moderate dementia in German DCCs. Evidence-based, non-pharmacological treatments are an effective addition to pharmacological interventions for individuals with cognitive impairment and help to improve the lives of these people. Owing to the limited resources in the health care system, decision makers can be supported by the knowledge of MAKS being a cost-effective intervention with low intervention costs. We recommend implementing MAKS as a regular non-pharmacological treatment in German DCCs. It can be supported financially in correspondence with the legal requirements of the German prevention law (§5, SGB XI) [87].

## Abbreviations

ADAS-Cog: Alzheimer’s Disease Assessment Scale—Cognitive Subscale
ADLs: Activities of daily living
ADCS-ADL: Alzheimer’s Disease Cooperative Study—Activities of Daily Living Inventory
BSFC-s: Burden Scale for Family Caregivers, short version
CEA: Cost-effectiveness analysis
CEAC: Cost-effectiveness acceptability curve
CE plane: Cost-effectiveness plane
CG: Control group
CI: 95% confidence interval
DCC: Day care center
DeTaMAKS-trial: German acronym for “*D*ementia in *D*ay care (German “*T*agespflege”) with *M*otor stimulation, *A*ctivities of daily living stimulation, *C*ognitive (German “*K*ognitiv”) stimulation, and *S*ocial functioning”
ETAM: Erlangen Test of Activities of Daily Living in Persons with Mild Dementia or Mild Cognitive Impairment
IG: Intervention group
ITT: Intention to treat
MAKS: Non-pharmacological treatment with four components—*M*otor stimulation, *A*ctivities of daily living stimulation, *C*ognitive stimulation, and *S*ocial functioning
MCI: Mild cognitive impairment
MMSE: Mini-Mental Status Examination
NOSGER: Nurses’ Observation Scale for Geriatric Patients, social behavior subscale
NPI-Q: Neuropsychiatric Inventory Questionnaire
SA: Sensitivity analysis
SD: Standard deviation
